# Integrating GWAS with gene expression data to dissect the genetic architecture of triple-negative breast cancer

**DOI:** 10.1186/gb-2011-12-s1-p35

**Published:** 2011-09-19

**Authors:** Chindo Hicks, Ranjit Kumar, Antonio Pannuti, Lucio Miele

**Affiliations:** 1 Cancer Institute, University of Mississippi Medical Center, 2500 North State Street, Jackson, MS 39216, USA

## Background

Research focused on genome-wide association studies (GWAS) has resulted in the identification of genetic variants associated with risk of developing breast cancer. These genetic variants are providing valuable insight into the genetic susceptibility landscape of breast cancer. However, to date, data generated from GWAS have not been maximally leveraged and integrated with gene expression data to identify the genes and pathways associated with the most aggressive subset of breast cancers, triple-negative breast cancer (TNBC), which accounts for about 20% of all breast cancers. TNBC disproportionately affects young premenopausal women and has a higher mortality rate among African-American women. At present, no targeted treatments exist for TNBC, and standard chemotherapy remains the only therapeutic option. Integration of genetic mapping results from GWAS with gene expression data could lead to a better understanding of the genetic mechanisms underlying the molecular basis of the TNBC phenotype and to the identification of potential biomarkers for the development of novel therapeutic strategies.

## Methods

We mined data from 43 GWAS involving over 250,000 patients with breast cancer and 250,000 controls, reported through April 2011, to identify genetic variants (single nucleotide polymorphisms (SNPs)) and genes associated with risk for breast cancer. We then integrated GWAS information with gene expression data from 305 subjects (162 cases and 143 controls) to stratify TNBC and other breast cancer subtypes, as well as to identify functionally related genes and multi-gene pathways enriched by SNPs that are associated with risk for breast cancer and are relevant to TNBC. To stratify TNBC and to identify functionally related genes, we performed supervised and unsupervised analysis of gene expression data. We used a false discovery rate to correct for multiple testing. Pathway prediction and networking visualization was performed using Ingenuity Systems’ software.

## Results

Combining GWAS information with gene expression data, we identified 448 functionally related genes that stratified breast cancer subtypes into TNBC. A subset of these genes (130 genes) contained SNPs associated with risk for breast cancer; of these 130 genes, 122 correctly stratified TNBC. Pathway prediction revealed multi-gene pathways enriched by SNPs that are significantly associated with risk for breast cancer. Key pathways identified include the p53, nuclear factor-κB, DNA repair and cell cycle regulation pathways.

## Conclusions

Our results demonstrate that integrating GWAS information with gene expression data can be an effective approach for identifying biological pathways that are relevant to TNBC. These could be potential targets for the development of novel therapeutic strategies.

